# Authentic Modeling of Human Respiratory Virus Infection in Human Pluripotent Stem Cell-Derived Lung Organoids

**DOI:** 10.1128/mBio.00723-19

**Published:** 2019-05-07

**Authors:** M. Porotto, M. Ferren, Y.-W. Chen, Y. Siu, N. Makhsous, B. Rima, T. Briese, A. L. Greninger, H.-W. Snoeck, A. Moscona

**Affiliations:** a Department of Pediatrics, Columbia University Medical Center, New York, New York, USA; b Center for Host-Pathogen Interaction, Columbia University Medical Center, New York, New York, USA; c Department of Experimental Medicine, University of Campania, Caserta, Italy; d Columbia Center for Human Development, Columbia University Medical Center, New York, New York, USA; e Columbia Center for Translational Immunology, Columbia University Medical Center, New York, New York, USA; f Department of Medicine, Columbia University Medical Center, New York, New York, USA; g Hastings Center for Pulmonary Research and Department of Medicine, Keck School of Medicine, University of Southern California, Los Angeles, California, USA; h Department of Microbiology and Immunology, Columbia University Medical Center, New York, New York, USA; i Department of Laboratory Medicine, University of Washington, Seattle, Washington, USA; j Vaccine and Infectious Diseases Division, Fred Hutchinson Cancer Research Center, Seattle, Washington, USA; k Center for Experimental Medicine, Queen's University, Belfast, Northern Ireland, United Kingdom; l Center for Infection and Immunity, Mailman School of Public Health, Columbia University, New York, New York, USA; m Department of Epidemiology, Mailman School of Public Health, Columbia University, New York, New York, USA; n Department of Physiology & Cellular Biophysics, Columbia University Medical Center, New York, New York, USA; St. Jude Children’s Research Hospital; University of Pittsburgh School of Medicine; Children's Hospital of Pittsburgh and University of Pittsburgh Medical Center

**Keywords:** lung organoids, parainfluenza virus, respiratory viruses, tissue infection model

## Abstract

Infectious viruses so precisely fit their hosts that the study of natural viral infection depends on host-specific mechanisms that affect viral infection. For human parainfluenza virus 3, a prevalent cause of lower respiratory tract disease in infants, circulating human viruses are genetically different from viruses grown in standard laboratory conditions; the surface glycoproteins that mediate host cell entry on circulating viruses are suited to the environment of the human lung and differ from those of viruses grown in cultured cells. Polarized human airway epithelium cultures have been used to represent the large, proximal airways of mature adult airways. Here we modeled respiratory virus infections that occur in children or infect the distal lung using lung organoids that represent the entire developing infant lung. These 3D lung organoids derived from human pluripotent stem cells contain mesoderm and pulmonary endoderm and develop into branching airway and alveolar structures. Whole-genome sequencing analysis of parainfluenza viruses replicating in the organoids showed maintenance of nucleotide identity, suggesting that no selective pressure is exerted on the virus in this tissue. Infection with parainfluenza virus led to viral shedding without morphological changes, while respiratory syncytial virus infection induced detachment and shedding of infected cells into the lung organoid lumens, reminiscent of parainfluenza and respiratory syncytial virus in human infant lungs. Measles virus infection, in contrast, induced syncytium formation. These human stem cell-derived lung organoids may serve as an authentic model for respiratory viral pathogenesis in the developing or infant lung, recapitulating respiratory viral infection in the host.

## Abstract (precis)

Respiratory viruses are among the first pathogens encountered by young children, and the significant impact of these viral infections on the developing lung is poorly understood. Circulating viruses are suited to the environment of the human lung and are different from those of viruses grown in cultured cells. We modeled respiratory virus infections that occur in children or infect the distal lung using lung organoids that represent the entire developing infant lung. These 3D lung organoids, derived from human pluripotent stem cells, develop into branching airway and alveolar structures and provide a tissue environment that maintains the authentic viral genome. The lung organoids can be genetically engineered prior to differentiation, thereby generating tissues bearing or lacking specific features that may be relevant to viral infection, a feature that may have utility for the study of host-pathogen interaction for a range of lung pathogens.

## INTRODUCTION

Paramyxoviruses and other RNA viruses are able to adapt remarkably rapidly to their host environment. They evolve nimbly under the selective pressure of viral inhibitors, immune attack, host tissue, or culture conditions; this flexibility allows paramyxoviruses to survive in a broad range of host environments. It has become evident that infectious viruses so precisely fit their hosts that the study of natural viral infection depends on host-specific mechanisms that affect virus-cell interplay. For the paramyxovirus human parainfluenza virus type 3 (HPIV3), for example, a prevalent cause of lower respiratory tract disease in children, circulating human viruses are genetically different from viruses grown in standard laboratory conditions (1). The surface glycoproteins on circulating viruses that mediate host cell entry are well suited to the natural environment of the lung and are different from the glycoproteins on the surfaces of viruses grown in immortalized monolayer cells. In fact, we recently showed that during even a brief adaptation to culture in immortalized cells, clinical viruses acquire genome-wide changes, resulting in alterations in the viral surface glycoproteins that mediate viral entry (2). These findings underscore the balance of features of the viral glycoprotein complex required for fitness in the human lung and the importance of studying infection in authentic tissues.

Acute respiratory infection is the leading cause of mortality in children under 5 years of age, accounting for one fifth of childhood deaths worldwide. Human parainfluenza viruses (HPIV) types 1, 2, and 3, human metapneumovirus, and respiratory syncytial virus (RSV) are enveloped negative-sense RNA viruses—paramyxoviruses and pneumoviruses—that cause the majority of childhood croup, bronchiolitis, and pneumonia. Despite the impact of these diseases on illness and hospitalization of young infants worldwide, no effective drugs or vaccines are available. Findings from our group and others have led to an understanding of the earliest stages of viral infection by HPIV3 that are paralleled in many respects in other paramyxoviruses, henipaviruses, and morbilliviruses (3–7), offering targets for prevention of viral entry and anti-infective therapy.

HPIV3 enters cells by fusing directly with the cell membrane. During entry, the viral surface glycoproteins HN (receptor binding protein; hemagglutinin-neuraminidase) and F (fusion protein) cooperate to form a complex that mediates fusion upon receptor binding. A series of coordinated interactions between HN and F glycoproteins, and a precise balance of the complex’s properties—receptor binding, receptor cleavage, activation of F protein by HN, triggering and insertion of F into the target, and stabilization of F—are critical for infection of a given host cell. The receptor binding protein HN, when engaged with the receptor, activates F to a fusion-ready state. Once activated, F inserts into the target membrane and undergoes a series of conformational changes that lead to fusion with the target host cell membrane. This paradigm for activation of F by HN has proven true across the negative-sense RNA virus paramyxoviruses, henipaviruses, and morbilliviruses. For RSV, a pneumovirus, the receptor binding protein (G) is responsible for engaging cellular receptors prior to fusion by F; however, while G-deficient viruses are severely handicapped *in vivo*, they can grow in immortalized monolayer cells (8). These differences in requirements for infection *in vivo* and *in vitro* illustrate the need for developing and studying authentic host tissue models of infection.

A polarized human airway epithelium (HAE) culture system has been used to represent authentic airway for respiratory virus infection. Primary HAE cells are cultured at an air-liquid interface, generating a differentiated, pseudostratified, mucociliary epithelium that faithfully represents the HAE (9). The HAE model was first used to characterize the polarity and cell specificity of respiratory syncytial virus (10, 11) and HPIV type 3 (HPIV3) (12–14), confirming that it is suited to studying paramyxovirus-pneumovirus-HAE interactions that reflect those in the human lung and a more physiological system than cell monolayers. Using HAE we have studied the differences between clinical strains of HPIV3 and laboratory-adapted viruses, after discovering that the fusion/entry complex of clinical isolates (CI) is significantly different from that of laboratory viruses (1, 2, 15). HAE provided a tissue environment that was sufficiently authentic so that no selective pressure was exerted on clinical strains of HPIV3 during growth in this system. In immortalized monolayer cell culture, even after only 3 to 4 days after infection, clinical strains evolve so that the fusion/entry complex acquires the traits needed for growth in culture and becomes less fit for the human lung (2). In contrast, in HAE, even after 7 days, the HN and F genes showed no evidence of evolution or adaptation to this tissue, establishing HAE as suitable for studying the properties of the fusion/entry complex required for fitness in the human lung (1, 2). However, the HAE model represents mainly the large, proximal airways and the mature, adult lung, and therefore has limitations in terms of representing respiratory viral infections. To address these issues, we explored modeling respiratory virus infection using organoids that are more representative of the entire lung and include other lung components. We recently reported that lung bud organoids derived from human pluripotent stem cells (hPSCs) contain mesoderm and pulmonary endoderm and develop into branching airway and early alveolar structures after plating in Matrigel (16). The tissues develop either in 3D culture or after xenotransplantation and thus far have attained the developmental stage of the end of the second trimester of human gestation. Infection of 3D lung organoid cultures with RSV led to detachment and shedding of infected cells into the lung organoid lumens, reminiscent of RSV disease in human infant lungs (16). hPSC-derived lung organoids therefore may serve as a model for respiratory viral infection in the developing or infant lung, complement the information gained from studies in HAE, and allow for study of infection in the distal lung, including the alveoli, which had not previously been possible. Here we investigate infection of hPSC-derived lung organoid cultures by HPIV3 and show that viral evolution and pathogenesis in the distal lung recapitulate important features of human viral infection.

## RESULTS

To characterize pathogenesis or molecular evolution of the HPIV3 fusion complex genes (HN/F) and in the entire genome of HPIV3 clinical isolates (CI), it is critical that viruses be propagated without adaptation to laboratory conditions in immortalized cells or providing selective pressure. We previously showed that clinical viruses can be propagated in HAE and preserve the features of the clinical virus entry complex (15, 17). Here we assessed whether the lung organoids provide an authentic representation of the human lung, supporting the growth of viruses in the absence of selective pressure for the virus to adapt to culture conditions. We asked whether propagation of CI in lung organoids preserves the features of the isolates or results in adaptation, as evidenced by alterations in the HPIV3 genome.

**FIG 1:**
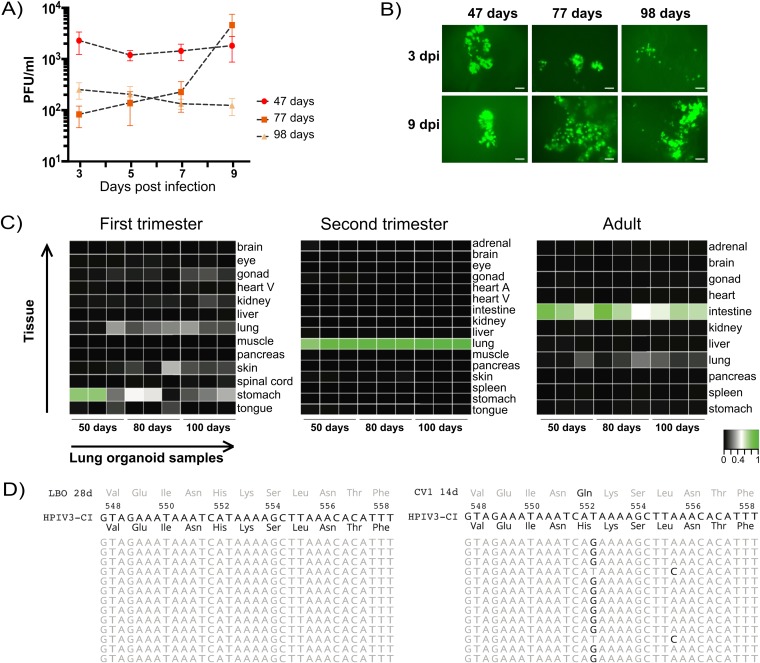
(A) Titer of HPIV3 CI after growth for 3, 5, 7, or 9 days in lung organoids that had been maintained for 47 days, 77 days, or 98 days. The experiment was performed three separate times, and the points represent PFU/ml ± SD. (B) Spread of recombinant CI expressing GFP in lung organoids after 3 days and 9 days of infection. dpi, days postinfection. Bars = 100 μm. (C) Representative RNA sequence data for the lung bud organoid tissues. Comparison of genome-wide expression in day 50, 80, or 100 lung organoids derived from hESCs with the KeyGenes database, showing the best match with second trimester human lung. (D) Conservation of clinical HPIV3 sequence in long-term LBO culture. In the left panel, deep sequencing reads of clinical HPIV3 grown in lung organoids revealed no genomic changes. In the right panel, after 14 days (14d) of culture in immortalized monolayer cells, a near clonal H552Q mutation (94% allele frequency) with an unlinked L555F minor allele (6%) was detectable. The mutated HN site II locus that has been identified as critical to tissue fitness (amino acids 548 to 558) is depicted.

Lung bud and subsequent branching lung organoid cultures were generated as reported previously (16) and as described in Materials and Methods. First, we determined the length of time for lung organoids in culture (the “culture age”) that supported infection and growth of HPIV3 and confirmed the culture age necessary to achieve the gene expression representative of late-second trimester human lung. For this purpose, organoids that had been cultured from day 0 for approximately 50, 80, or 100 days were infected with either clinical isolate from a patient sample (CI-1 [1, 15]) or recombinant HPIV3 expressing enhanced green fluorescent protein (eGFP) for ease of tissue detection (10). Tissues were either collected for viral titering or processed for RNA sequencing. Figure 1A shows the titers of HPIV3 CI and HPIV3rec-eGFP after growth for 3, 5, 7, or 9 days in organoids that had been maintained for 47 days, 77 days, or 98 days. The experiment was performed three separate times (three organoids from three separate batches for each time point), and the points represent PFU/ml ± SD. For these collections, supernatant fluid was harvested from the small amount of feeding medium on top of the Matrigel insert. In Fig. 1B, the spread of recombinant GFP-expressing CI through the lung organoids at day 3 and day 9 after infection can be seen. Figure 1C shows the representative RNA sequence data for the lung organoid tissues. Comparison of genome-wide expression in day 50, 80, and 100 lung organoids using the KeyGenes database revealed that all three lung organoid cultures were an excellent match with second trimester human lung (18). Thus, even tissues cultured for as little as 47 days supported growth of HPIV3 clinical and recombinant viruses and accurately represent late-second trimester human lung. The possibility of working with these cultures after a relatively short period of time in culture increases their potential utility.

To determine whether the CI viruses propagated in lung organoids preserve their genome sequences or show evidence of selective pressure, we analyzed the whole-genome sequences of the viruses collected during lung organoid infections by NGS and compared them to the sequences of the same viruses grown in parallel in either HAE or immortalized monolayer CV1 cells. Only three mutations—two of which were synonymous—occurred in the entire HPIV3 genome during passage in HAE or lung organoid cultures. These mutations were present in around 40 to 60% of the reads whereas they were present in below 10% of the reads in the clinical strain input. They occurred at positions 6207 (a synonymous mutation in the F gene) and 6338 (a nonsynonymous mutation in the HN protein changing amino acid 379 from valine to alanine), and there was a synonymous mutation in the L gene at position 11489. Even after 28 days of infection, CI HPIV3 passaged in lung organoid cultures showed no genomic changes compared to fixation of HN H552Q in CV1 cells at day 14 in culture. The sequence data in Fig. 1D focus on a specific region in HN that undergoes rapid change when clinical HPIV3 adapts to growth outside the lung tissue (2) and show deep sequencing reads of HPIV3 CI grown in lung organoids on the left and in an immortalized cell line (CV1) on the right. In lung organoids, no genomic changes were observed. In contrast, as expected, after 14 days in immortalized cells, a near clonal H552Q mutation (94% allele frequency) with an unlinked L555F minor allele (6%) was detected. Similar to what we previously observed for HAE, and in contrast to culture on immortalized monolayer cells (1, 2, 17), the viral genome is thus preserved during growth in lung organoids, indicating that the selective environment is representative of human lung.

**FIG 2:**
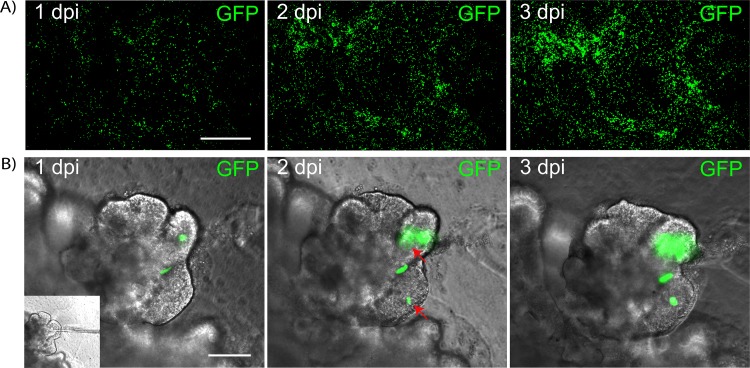
(A) Representative images showing the spread of recombinant HPIV3-GFP in HAE and lung organoids on days 1, 2, and 3 after infection. Bar, 100 μm. (B) Localized recombinant HPIV3-GFP infection and the spread in lung organoids over time. Bar, 100 μm.

HPIV3 viral spread in infected HAE and lung organoids was compared using recombinant HPIV3 expressing GFP (Fig. 2). HPIV3 spreads evenly throughout HAE tissue over the first 3 days of infection as evidenced by the spread of green fluorescence (Fig. 2A). In the lung organoid, the spread of infection can be seen in Fig. 2B within an alveolar structure. Contiguous spread is evident as enlargement of one site of infection at day 1, and spread to other nearby parts of the structure can be clearly seen by day 3 (red arrows). These findings are consistent with authentic infection at several sites followed by more distant spread as released particles can move through the structure. We infected organoids either by depositing the inoculum beneath the Matrigel layer adjacent to the organoid tissue or by microinjecting the inoculum directly into the alveolar space and identified little difference in initial infection or spread with these two methods, suggesting that the virus is targeted to the alveolar lining by either approach. Figure S1 in the supplemental material shows microinjection of dye into the lumen and spread of dye within the alveolar structure, demonstrating the patency of this space. Establishment of infection in the organoid was similar by either method as determined by immunofluorescent staining for viral antigen or spread of GFP-expressing recombinant viruses (data not shown).

10.1128/mBio.00723-19.1 FIG S1 Patency of the lung organoid alveolar structure. FITC-dextran dye was microinjected into the lung organoid structure with low pressure, and diffusion of the dye was monitored over time (times shown, 5 min, 7 min, and 32 min). Download FIG S1, TIF file, 16.0 MB. Copyright © 2019 Porotto et al. 2019 Porotto et al. This content is distributed under the terms of the Creative Commons Attribution 4.0 International license.

**FIG 3:**
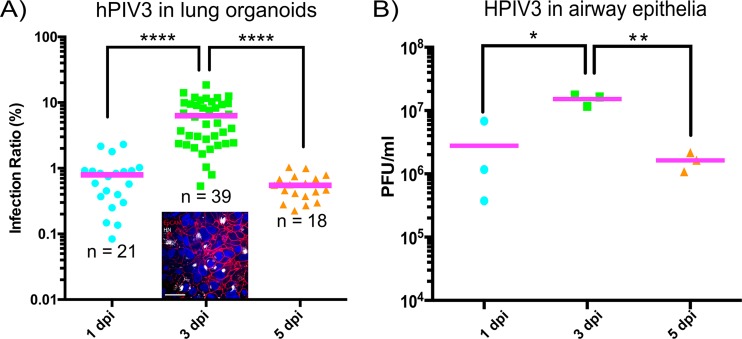
Comparison of peak infection timing in lung organoids and HAE. (A) Infection intensity in the dot plot measured by counting the ratio of HN-positive pixels to DAPI-positive pixels in representative confocal images from whole-mount immunostaining of lung organoids infected with HPIV3-CI. (Inset) Representative image used to calculate this value, with viral HN antigen (white) epithelial cell adhesion molecule (EpCAM) outlining alveolar epithelial cells (red) and DAPI nuclear stain (blue). Bar = 25 μm. (B) Growth curve of HPIV3 CI represented by viral titer in the HAE supernatant fluid. Values that are significantly different by two-way ANOVA (*n* = 3 or more) are indicated by bars and asterisks as follows: *, *P* < 0.05; **, *P* < 0.01; ****, *P* < 0.0001.

The timing of peak infection was surprisingly similar in lung organoids and HAE, with peak infection of airway cells in both models occurring at 3 days after infection and beginning to decline by day 5. While we can determine titers of virus collected from the feeding medium on top of the organoids’ Matrigel insert (as in Fig. 1), these measurements are not comparable in a quantitative way with titers from HAE media. Therefore, we assessed the timing of infection in organoids and HAE using methods that are precise and internally consistent for each system. The dot plot in Fig. 3A shows the “infection intensity” of the organoids on days 1, 3, and 5 postinfection, as measured by counting the ratio of HN-positive pixels to DAPI-positive pixels in representative confocal images from whole-mount immunostaining of lung organoids infected with HPIV3-CI. The inset shows a representative image used to calculate this value. For HAE, Fig. 3B shows the growth curve of HPIV3-CI represented by viral titer in the HAE supernatant fluid at the same time points.

**FIG 4:**
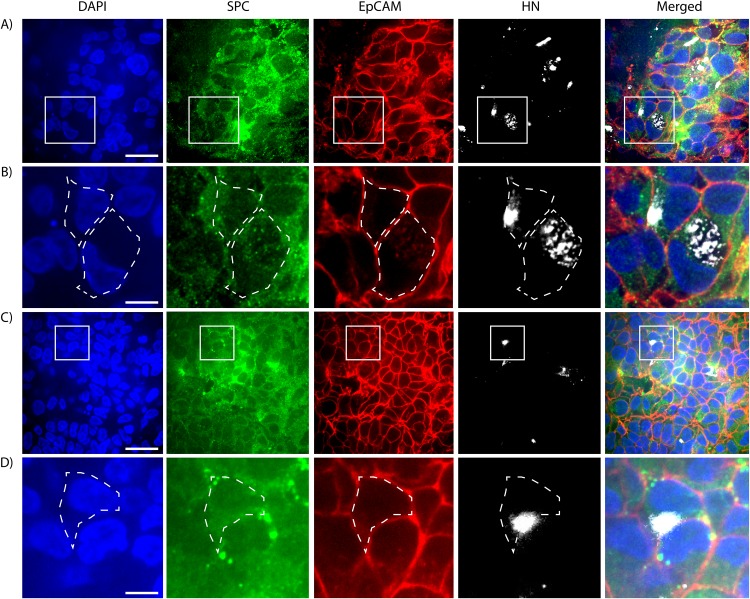
HPIV3 (clinical isolate) infects type II lung alveolar epithelial cells in the lung organoids. (A and C) Two representative images showing the colocalization of immunostaining signals for viral HN antigen (white) and the type II alveolar epithelial cell marker SPC (green) on single cells. Bars = 25 μm. Zoomed-in areas of the micrographs are outlined by white squares. (B) Zoomed-in images of the areas outlined in panel A; representative infected single cells are outlined with white dashed lines. Bar = 6.25 μm. (D) Zoomed-in images of the area outlined in panel C; representative infected single cells are outlined with white dashed lines. Bar = 6.25 μm.

Representation of the distal airway structures, including alveolar cell types, makes the lung organoid a potentially important complement to HAE for respiratory virus pathogenesis studies. HPIV3 CI infect type II alveolar epithelial cells in this model, as shown in Fig. 4. Two representative confocal images from whole-mount immunostaining of infected lung organoid, each with a zoomed-in counterpart, are shown with individual immunofluorescent staining and a merged image at the far right. DAPI nuclear stain is shown in blue, pulmonary surfactant-associated protein C (SPC) to identify type II alveolar epithelium cells is shown in green, epithelial cell adhesion molecule (EpCAM) to outline the alveolar epithelial cells is shown in red, and anti-HN antibodies are used to locate viral antigen is shown in white in Fig. 4.

**FIG 5:**
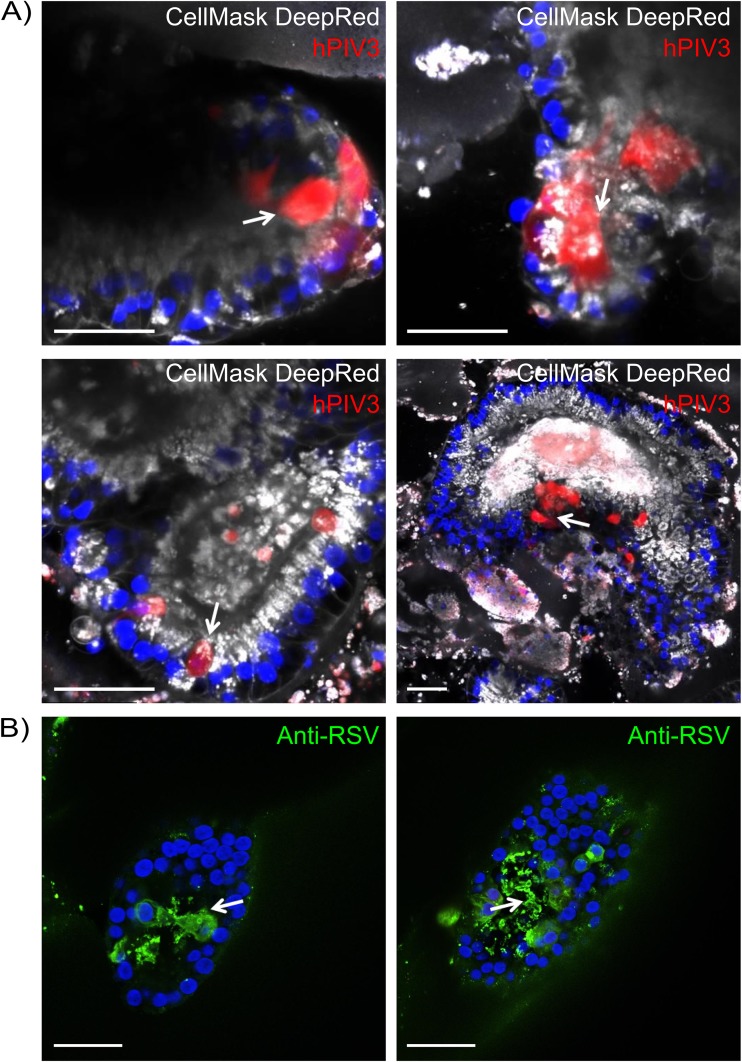
Confocal images of whole-mount lung organoids 32 days after infection with recombinant CI hPIV3 or RSV. In the hPIV3-infected organoids, the GFP-expressing virus is shown in red, and membrane staining using CellMask Deep Red plasma membrane stain (catalog no. C10046; Thermo Fisher) is shown in white. The RSV in infected organoids are stained with anti-RSV antibody (green) and similar to images we published in reference 16. Bars = 50 μm.

To explore whether lung organoids infected with HPIV3 display 3D features of human lung infection, we examined whole-mount tissues by confocal microscopy. Previous studies have suggested that in contrast to RSV, where infected cells swell and detach from the epithelium, HPIV3-infected cells are not shed (11). Clinically, HPIV3 disease is less marked by small airway obstruction than RSV disease (19, 20). At day 32 after infection of day 170 lung organoid cultures with HPIV3, confocal microscopy revealed infected cells surrounding the lumen of the branching structures (Fig. 5A), and the greater extent of infection could be seen (Fig. 5B). However, unlike what we had observed for RSV (16), there was no detectable change in tissue integrity or shedding of infected cells into the lumen despite evidence of viral infection, consistent with the clinical observations for HPIV3 infection.

**FIG 6:**
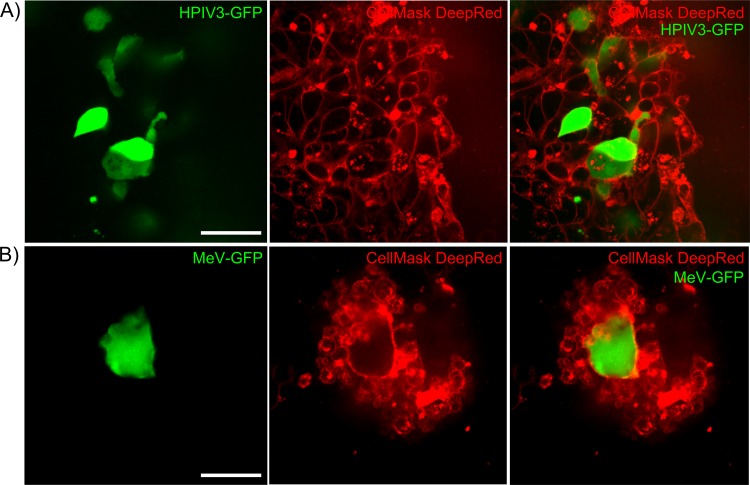
**(**A) Representative image of recombinant HPIV3 infection in lung organoid model 2 days after infection. Infected cells express GFP (green), and cellular membranes are delineated using membrane dye (red). Neither syncytium formation nor obvious cytopathology was observed. Bar = 40 μm. (B) Representative image of recombinant measles virus infection in lung organoid model at 2 days after infection. Infected cells express GFP (green), and cellular membranes are delineated using membrane dye (red). A multinucleated syncytium contains multiple infected lung organoid cells. Bar = 40 μm.

To address whether the lung organoid model may serve in the future to compare and differentiate modes of infection between important viruses that infect the respiratory tract, we compared HPIV3 infection with measles virus (MeV) infection. MeV is known to infect respiratory tissue via the basolateral surface in distinction to HPIV3, which reaches the apical epithelial cells to infect (10, 13), and in distinction to HPIV3, MeV infection induces syncytium formation even in healthy hosts (21). Recombinant viruses expressing GFP were employed for both HPIV3 and MeV for ease of comparison in 3D lung organoids (Fig. 6), and membrane dye was used to delineate cell borders for imaging within the tissue. Many HPIV3-infected cells are evident in Fig. 6A (top panels) at 2 days after infection, and the merged image shows contained, circumscribed foci of viral entry without syncytium formation. In contrast, Fig. 6B (bottom panels) shows syncytium formation caused by MeV at 2 days after infection. These differences are even more remarkable in movies obtained from z-stacking deconvolution that travel the full thickness of the tissue where large areas of fusion involving multiple cells are seen in the MeV-infected tissues, while individual membranes of cells in the HPIV3-infected tissues remain evident (see Movies S1 and S2 in the supplemental material).

10.1128/mBio.00723-19.2 MOVIE S1 3D scan of HPIV3-infected lung organoid showing pattern of infection without syncytium formation. GFP from recombinant GFP-expressing recombinant HPIV3 indicates viral infection; red membrane dye highlights the cell borders as for Fig. 6. Download Movie S1, MOV file, 0.4 MB. Copyright © 2019 Porotto et al. 2019 Porotto et al. This content is distributed under the terms of the Creative Commons Attribution 4.0 International license.

10.1128/mBio.00723-19.3 MOVIE S2 3D scan of MeV-infected lung organoid showing pattern of infection with giant multinucleated syncytium formation, most notable at ∼4 s. GFP from recombinant GFP-expressing recombinant MeV indicates viral infection; red membrane dye highlights the cell borders as for Fig. 6 and Movie S1. Download Movie S2, MOV file, 0.5 MB. Copyright © 2019 Porotto et al. 2019 Porotto et al. This content is distributed under the terms of the Creative Commons Attribution 4.0 International license.

## DISCUSSION

Viruses tailor themselves for fitness in their hosts with great specificity. For respiratory viruses, molecular evolution, fitness, and pathogenesis are in direct interplay with the host and can be ideally studied under conditions where these features are authentic. The hPSC-derived lung organoid model, in which lung bud organoids are embedded in Matrigel for development after day 25, offers a complement to adult HAE for studies of respiratory viral infection. In our model, clinical viruses preserve their whole-genome sequence and therefore appear to be under no particular selective pressure, unlike clinical viruses grown in immortalized cells where the viruses rapidly evolve a new fusion complex phenotype (2). Viral growth is supported in a time line that mirrors viral replication in humans, with infection of alveolar epithelial cells in a pattern like that of HPIV3 infection *in vivo*. Comparison of HPIV3 infection and RSV infection that we previously reported (16) as well as a head-to-head comparison in 3D of HPIV3 and MeV infection indicates that respiratory viral infection in lung organoids recapitulates important differences between viral respiratory infections in humans.

The lung organoids are a developmental model in contrast to the fully mature HAE, so that the former offers the opportunity to assess infection at different stages of development. For example, infection of lungs at different degrees of prematurity can be represented, and the organoid model offers the opportunity to explore how early viral infection may impact subsequent lung development. Lung organoids and HAE also contain distinct lung cell types; the organoids represent more distal airways than the HAE, which are a more accurate representation of the large proximal airway and lack certain cell types that may be important for viral infection (e.g., Clara cells) (11). A current limitation of this model is that the lung organoids have not yet been induced to proceed in development beyond the end of the second trimester human lung. Efforts are under way to model later stages of lung development.

As a useful future complement to the lung organoid system described here using Matrigel to embed the developing organoid, we have shown that when transplanted under the kidney capsule of highly immunodeficient NOD.Cg-Prkdcscid.Il2rgtm1Wjl/SzJ (NSG) mice, lung bud organoids also yielded branching structures representative of late-second trimester human lung. This xenotransplantation model would offer the possibility of defining the host immune system by manipulating the host mice, thereby adding complexity (e.g., human immune system grafting), and this will be explored in future studies.

The finding that infection of lung organoids recapitulates some of the hallmarks of illness for each respiratory virus we tested (e.g., epithelial sloughing and obstructed airways for RSV-infected cells versus no sloughing for HPIV3-infected cells) suggests that this model will be useful for studying pathogenesis; for example, it can be used to test hypotheses about the contribution of specific components of the human immune system to pathogenesis for each virus. For MeV, infection of airway epithelial cells is postulated to occur only after the initial viremia and to involve cell-to-cell contact between infected myeloid cells and epithelial cells (14) in the nonhuman primate model, though this is difficult to observe directly in studies involving human subjects (22). While the current lung organoid model cannot yet recapitulate these elements, interestingly in contrast with MeV in HAE (13, 23) and HPIV3 infection in the lung organoid model, MeV induces syncytia in the lung organoid model (most clearly seen 4 s into Movie S2 in the supplemental material). This resembles the characteristic cytopathology caused by MeV in humans and is in marked contrast to what has been observed in HAE (14, 24). The organoid model may thus be useful in elucidating how MV is shed.

Investigation of host cell factors important for respiratory viral infection has been hindered by the scarcity of relevant physiologically authentic lung models. Certainly, given what we now know about the selective pressure placed on viruses by immortalized monolayer cell culture systems, these cannot be considered informative with respect to host factors. The polarized HAE culture seems to provide the necessary host factors to avoid selective pressure, and this system has been informative for the study of biologically relevant features of the HN/F entry complex and requirements for infection. The utility of HAE for identification of important host factors for entry has, however, been limited by the fact that they are not amenable to genetic manipulation because they are derived from adult donors and are fully differentiated. It is not possible to guarantee homogeneity of the HAE tissues, and it is not possible to specifically engineer desired host features—elements of innate immunity, processing enzymes involved in viral infection, or even underlying human genetic diseases. As the lung organoids are derived from either human embryonic stem cells (hESC) or from induced pluripotent stem cells (iPSCs), they can be genetically engineered prior to differentiation (16), thereby generating tissues bearing or lacking specific features that may be relevant to viral infection. We anticipate that this may have great utility for the study of infection in the lung and host-pathogen interaction for a range of lung pathogens.

## MATERIALS AND METHODS

### HPIV3 strains.

HPIV3 clinical isolate virus (CI-1) was obtained from the Clinical Microbiology Laboratories at New York Presbyterian Hospital as a deidentified aliquot of the original sample material and grown in human airway epithelium (HAE) at an air-liquid interface for only one passage prior to use in these experiments. Laboratory-adapted recombinant viruses expressing GFP were obtained from Ursula Buchholz and Peter Collins (NIAID) (10).

### Recombinant measles virus.

MeV IC323-EGFP (25) is a recombinant virus generated by reverse genetics to express the gene encoding EGFP (using the plasmid encoding MeV IC323-EGFP kindly provided by Yusuke Yanagi, Kyushu University, Fukuoka, Japan). MeV IC323 recombinant viruses were rescued in 293-3-46 cells as previously described (26). All viruses were propagated, and virus titers were determined in Vero-SLAM cells.

### Cells.

CV1 (African green monkey kidney), 293T (human kidney epithelial), 293-3-46 (26), Vero, and Vero-SLAM (African green monkey kidney) cells were grown in Dulbecco’s modified Eagle’s medium (DMEM) (Invitrogen; Thermo Fisher Scientific) supplemented with 10% fetal bovine serum (FBS) and antibiotics in 5% CO_2_. The 293-3-46 and Vero-SLAM culture media were supplemented with Geneticin (1 mg/ml) (Thermo Fisher Scientific).

### HAE cultures.

The EpiAirway AIR-100 system (MatTek Corporation) consists of normal human-derived tracheo/bronchial epithelial cells that have been cultured to form a pseudostratified, highly differentiated mucociliary epithelium closely resembling that of epithelial tissue *in vivo*. Upon receipt from the manufacturer, HAE cultures were transferred to six-well plates (containing 0.9 ml medium per well) with the apical surface remaining exposed to air and incubated at 37°C in 5% CO_2_.

### Lung bud organoid cultures.

The cultures were generated and differentiated as described in reference 16 and summarized briefly below. Maintenance medium consisted of DMEM/F12 (1:1) supplemented with 20% knockout serum replacement, 0.1 mM β-mercaptoethanol, Primocin, and 20 ng/ml FGF-2. Serum-free differentiation (SFD) medium consisted of IMDM/Ham’s F12 (3:1) supplemented with N2, B27, 0.05% bovine serum albumin, 1% penicillin-streptomycin, 50 μg/ml ascorbic acid, 2 mM Glutamax, 0.4 μM monothioglycerol, and growth factor cocktails as indicated in reference 16.

Rockefeller University embryonic stem cell line 2 (RUES2, NIH approval number NIHhESC-09-0013, Registration number 0013, passage 17 to 28) were maintained on mouse embryonic fibroblasts (MEFs) plated at 15,000 to 18,000 cells/cm^2^. Cells were cultured in maintenance medium, and medium was changed daily. Cultures were maintained in a humidified 5% CO_2_ atmosphere at 37°C. Lines are karyotyped and verified for *Mycoplasma* contamination using PCR every 6 months.

**(i) Endoderm induction.** Induction of endoderm was conducted as previously described (27). Briefly, MEFs were depleted by passaging onto Matrigel for 24 h supplied with maintenance medium and maintained in a humidified 5% CO_2_ atmosphere at 37°C. After MEF depletion, primitive streak and embryoid body induction was performed in embryoid bodies/primitive streak formation media in low-attachment plates for 12 to 16 h followed by switching to endoderm induction medium for 36 to 40 h. Embryoid bodies were fed every day and maintained in a humidified 5% CO_2_/5% O_2_ atmosphere at 37°C. Endoderm yield was determined by the expression of CXCR4 and c-KIT. For iPS lines, endodermal cells were purified using human CD184 (CXCR4) MicroBead kit. Cells used in all experiments had >90% endoderm yield.

**(ii) Anterior foregut endoderm induction.** Anterior foregut endoderm was induced as previously described (27). On day 4, embryoid bodies were dissociated with 0.05% trypsin/EDTA and plated on fibronectin-coated multiple-well plates with a density at 80,000 to 105,000 cells/cm^2^. Cells were incubated in anteriorization medium 1 for 24 h, followed by switching to anteriorization medium 2 for another 24 h.

**(iii) Formation of lung bud organoids.** At the end of anterior foregut endoderm induction, cells were treated with ventralization medium (27) for 48 h, and three-dimensional clump formation was observed. The clumps were then suspended by gently pipetting around the wells. The suspended clumps were called lung bud organoids (LBOs) hereafter. LBOs were maintained in non-tissue culture-treated multiple-well plates submerged in branching medium and were fed every other day until day 20 to day 25.

**(iv) Branching morphogenesis in Matrigel.** The day 20 to day 25 LBOs were embedded in 100% Matrigel in 24-well transwell inserts and incubated in an incubator until the Matrigel solidified. Branching medium was added to the well, after which the transwell was inserted; branching medium was added into the transwell insert as well. The medium was changed every other day. A step-by-step protocol describing the generation of LBOs and LBO-derived branching colonies in Matrigel can be found at Nature Protocol Exchange (27).

**(v) Immunofluorescence staining.** Lung organoid and branching Matrigel cultures were freshly embedded in OCT. Samples were sectioned between 5 and 8 μm and then air dried for 2 h. The sections were fixed with 4% paraformaldehyde for 20 min at room temperature (RT) and washed with DPBS for 5 min. The sections were permeabilized with 0.3% Triton X-100/PBS for 30 min, followed by blocking in 5% donkey serum for 1 h. Primary antibodies (as indicated in the figure legends) were incubated at 4°C overnight. The next day, the sections were washed with DPBS three times for 5 min each time followed by secondary antibody incubation for 2 h at RT, washed three times for 10 min each time with DPBS, and then mounted with DAPI-containing fluorescent mounting medium.

### RNAseq.

Total RNA from lung organoids was purified using Direct-zol RNA MicroPrep kit. RNA concentration and RNA integrity number (RIN) were determined using an Agilent microfluidic RNA 6000 Nano Chip kit (Agilent Technologies, Santa Clara, CA) on the 2100 Bioanalyzer (Agilent Technologies, Santa Clara, CA). Those samples with RIN greater than 9 were used for RNAseq. Poly(A) pulldown was used to enrich mRNAs from total RNA samples. Libraries were prepared using Illumina TruSeq RNA prep kit (Illumina, San Diego, CA). Libraries were then sequenced using the Illumina HiSeq2000 (Illumina, San Diego, CA) at the Columbia Genome Center. Samples were multiplexed in each lane, yielding a targeted number of single-end/pair-end 100-bp reads for each sample, as a fraction of 180 million reads for the whole lane. RTA (Illumina, San Diego, CA) was used for base calling, and bc12fastq (version 1.8.4) was used to converting BCL to fastq format, coupled with adaptor trimming. Reads were mapped to a reference genome (NCBI/build37.2) using Tophat (version 2.0.4) with 4 mismatches and 10 maximum multiple hits. To tackle the mapping of reads that are from exon-exon junctions, Tophat infers novel exon-exon junctions *ab initio* and combines them with junctions from known mRNA sequences as the reference annotation. We estimated the relative abundance of genes and splice isoforms using cufflinks (version 2.0.2) with default settings. We tested for differentially expressed genes under various conditions using DEseq, an R package based on a negative binomial distribution that models the number reads from RNAseq experiments and tests for differential expression.

### Comparative analysis using KeyGenes.

RNAseq data obtained from day 50, day 80, and day 100 lung organoids from RUES2 was compared to different first and second trimesters and adult organs, including the lungs, using KeyGenes. Hierarchical clustering of our laboratory samples and 75 samples from 19 organs from second trimester was performed using Cluster 3.0 and viewed by TreeView. The 87 classifier genes were calculated by KeyGenes.

### Viral propagation in HAE.

HAE cultures were infected by applying 200 μl of EpiAirway phosphate-buffered saline containing 4,000 PFU of reference or other strain of HPIV3 to the apical surface for 90 min at 37°C. At 90 min, the medium containing the inoculum was removed, and cultures were placed at 37°C and fed each day with 0.9 ml medium via the basolateral surface. Viruses were harvested by adding 200 μl medium per well to the HAE cultures’ apical surface and allowed to equilibrate for 30 min at 37°C. The suspension was then collected, and viral titers were determined as previously described (28). This viral collection was performed sequentially with the same wells of cells on each day postinfection.

### Preparation and sequencing of HPIV3 RNA.

Clinical isolates, along with laboratory-adapted virus, were all grown in either HAE, CV1 cells, or lung organoids. RNA was extracted and sequenced from extracellular supernatant fluid.

### Genome assembly.

Reference-guided genome consensus assemblies were generated for each sample by aligning the sequencing reads to HPIV3 strain 14072 (GenBank accession no. EU424062.1) using BWA-MEM v0.7.5a-r405, removing multimapped reads, and resolving conflicts using a majority rule. (Priority was given in the order A > C > G > T in the event of a tie.) Sequence coverage was determined using the SAMtools depth tool v1.1 (29). mNGS libraries for day 28 lung organoids and day 14 CV1 passage experiments were prepared as described previously (30). RNA was extracted from viral supernatant using the Zymo Viral RNA kit and treated with Turbo DNase I at 37°C for 20 min (Thermo Fisher). RNA was reverse transcribed using random hexamers SuperScript IV (Thermo Fisher), and double-stranded cDNA was produced using the same random hexamers with Sequenase 2.0 (Thermo Fisher) and cleaned using a Zymo DNA Clean and Concentrator. Sequencing libraries were generated using one-third volumes of Nextera XT tagmentation followed by 20 cycles of dual-indexed PCR and a 0.8X Ampure bead cleanup (Beckman Coulter). Libraries were sequenced on an Illumina MiSeq. Sequencing reads are deposited in NCBI under BioProject PRJNA524147.
