# Supplementary figures and images for: Authentic Modeling of Human Respiratory Virus Infection in Human Pluripotent Stem Cell-Derived Lung Organoids
Source: mBio. 2019 May 7;10(3):e00723-19. doi: 10.1128/mBio.00723-19 (PMC6509192; doi:10.1128/mBio.00723-19)

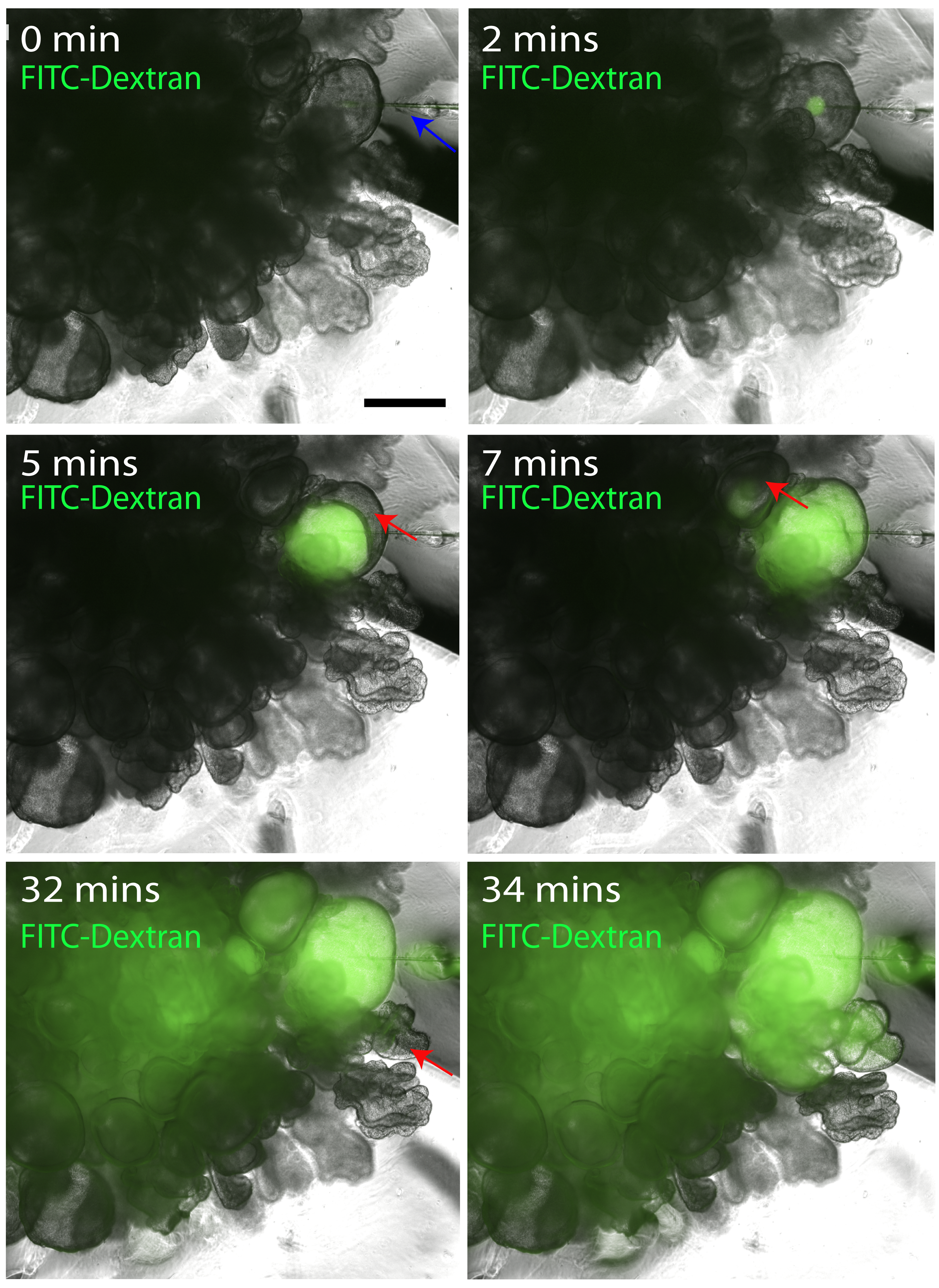

Supplement: FIG S1 [file mBio.00723-19-sf001.tif]
